# Care of family caregivers of persons with dementia (CaFCa) through a tailor-made mobile app: study protocol of a complex intervention study

**DOI:** 10.1186/s12877-020-01712-7

**Published:** 2020-08-26

**Authors:** Zarina Nahar Kabir, Angela Yee Man Leung, Åke Grundberg, Anne-Marie Boström, Kristina Lämås, Ana Paula Kallström, Cecilia Moberg, Berit Seiger Cronfalk, Sebastiaan Meijer, Hanne Konradsen

**Affiliations:** 1 Division of Nursing, Department of Neurobiology, Care Sciences and Society, Karolinska Institute, Alfred Nobels Allé 23, 141 83 Huddinge, Stockholm, Sweden; 2 Centre for Gerontological Nursing, School of Nursing, The Hong Kong Polytechnic University, Hong Kong, Hong Kong; 3 Department of Nursing Science, Sophiahemmet University, Stockholm, Sweden; 4 Theme Aging, Karolinska University Hospital, Huddinge, Sweden; 5 R&D unit, Stockholms Sjukhem, Stockholm, Sweden; 6 Department of Nursing, Umeå University, Umeå, Sweden; 7 Division of Nursing, Red Cross University College, Stockholm, Sweden; 8 Royal Institute of Technology, Stockholm, Sweden; 9 Herlev and Gentofte Hospital, Department of Gastroenterology, Copenhagen, Denmark; 10 Department of Clinical Medicine, Faculty of Health and Medical Sciences, University of Copenhagen, Copenhagen, Denmark

**Keywords:** Caregiver stress, Caregiving, Depressive symptoms, Family caregiver, Loneliness, mHealth, Person with dementia, Telehealth

## Abstract

**Background:**

Globally, family members account for the main source of caregiving of persons with dementia living at home. Providing care to family members with dementia often has negative health consequences for caregivers such as stress, depression and low quality of life. Yet, formal support for family caregivers (FCs) is limited. Telehealth technology has the potential to provide health care and social support to FCs. This study aims to assess the effectiveness of providing support by healthcare professionals (HPs) through a mobile app in reducing stress, depressive symptoms and loneliness, and improving mental health and quality of life of FCs of persons with dementia.

**Methods:**

Using a pragmatic intervention design, this study will use pre- and post-intervention assessment to evaluate the effectiveness of the proposed intervention in a sample of 78 FCs of persons with dementia (PWD). The intervention will be implemented by approximately 5 HPs specialized in dementia care based in the municipalities in Sweden. The main thrust of the intervention is to provide professional support, with help of an interactive mobile app, to family members in their caregiving role for PWDs. Qualitative interviews with HPs and FCs form the groundwork of the development of the mobile app. By using the app on smart phone or tablet, the FC, in groups of 8–10, will communicate with peers and a HP exchanging ideas on how to deal with PWD’s behavioral and cognitive changes and get support. They will also be able to discuss stressful events and access mindfulness exercises focused on themselves. Quantitative data will be collected before and at three time points after the 8-week intervention to assess changes in the health outcomes of the FCs. In-depth interviews will be conducted after the intervention to capture the experiences of FCs and HPs regarding the ease of use and acceptability of the app.

**Discussion:**

This tailor-made mobile app has the high potential to be a practical platform for supporting FCs to alleviate stress and improve mental health irrespective of distance to the nearest health care or social service center.

**Trial registration:**

ISRCTN, ISRCTN46137262. Registered 10 October 2019.

## Background

Family members are the main source of care for persons with dementia (PWD) living at home although their accessibility and dependence on them may vary among high-, middle- and low-income countries [1]. In 2015, approximately 6 h was spent daily by caregivers (71% women) in caring for a PWD in the family, globally [2]. There are over 100 million unpaid caregivers in Europe whose contribution to the economy is significant. According to the latest available statistics in Sweden from 2012, one in five adults provides care to a family member in need of help and this is estimated to be equivalent to more than one million in total. More than one third of these family caregivers are aged 65 years or older [3].

### Health consequences of family caregiving

Although most caregivers are positive about the experience of caregiving, it is challenging to provide 24-h caregiving. Thus many caregivers suffer from stress and depression [2]. A review of 37 studies on health consequences of being caregivers to family members with dementia found a majority of caregivers were over 60 years old and living in a state similar to chronic stress [4]. The majority of spousal caregivers have health and well-being problems of their own, either pre-existing or arising from the role of caregiving activities, e.g. due to the tasks of caring and the distress associated with the role [5]. Stress of a family caregiver may even reach a point that the person cared for may be transferred to a nursing home [6].

Caregivers are described to be at high risk of depression and anxiety due to their caregiving role and related responsibility [7, 8]. Many family caregivers of PWD report being highly burdened (68%) and having depressive symptoms (65%) [9]. Depression, as consequence of caregiving to a family member with dementia [10], may remain even after institutionalization or death of the care recipient [11]. The intensity of caregiving is reported to be inversely related to the caregiver’s quality of life [3]. It is also affected through a sense of isolation and loneliness.

### Support for family caregivers of PWD

At the early stage of dementia, PWD and family caregivers may not come in contact with the health care or socials services. Consequently, the social welfare system has no indication of what professional support is needed by the patients and their family members [12]. Support with daily living, navigating the healthcare system, emotional support, and coping strategies are important issues for family caregivers. Research from Sweden suggests adoption of person-centred approach to meet individual needs of family caregivers of PWD who experience lack of support in dealing with the behavioural and cognitive changes in the PWD that they care for, and their own sense of vulnerability and isolation [13].

### Technology-based interventions to support caregivers of older adults

Telehealth technology can support caregivers, facilitate significant improvements in caregivers’ coping, and positively affect care of chronic disease in the home as it combines elements of education, consultation, psychosocial therapy, social support and clinical care [14]. A review of internet-based interventions for caregivers of older adults concluded that interventions incorporating professional and social support with interactive ways of providing instructions on behavioral change and problem solving can lead to positive outcomes for caregivers [15].

In recent years, the use of information and communication technology (ICT) has opened novel opportunities to support caregivers when traditional face-to-face formats are hindered by geographical barriers or community resources. Examples of ICT interventions for supporting caregivers of PWD include RHAPSODY (Research to Assess Policies and Strategies for Dementia in the Young) project, an European initiative which provided internet-based information and skill-building program for family caregivers [16]; Diapason, a web-based fully automated psychoeducational program for informal caregivers of patients with Alzheimer’s Disease [17]; a recent study titled “Health e-Brain” which focuses on providing support to technology-enabled caregivers of persons with Alzheimer’s Disease through a protocol delivered through a web portal of the study [18]. These web-based dementia caregiving programmes initiated a new direction for supporting caregivers and reducing their stress. The RHAPSODY project reports acceptance by caregivers of web-based interventions and positive outcome on reducing their stress [19]. A short-term telephone based individualised intervention programme using cognitive behavioural therapy approach found long-term effects on emotional well-being, health status, bodily complaints and quality of life of family caregivers of PWD [20]. However, web-based intervention is not always optimal for caregivers due to the limitations of web layout and the restriction of frequency of updating. Also, the existing studies focused on individuals but had not taken into account how caregivers can support each other in their caregiving roles and even as support for each other. With an increasing older population in Sweden, the caregivers of older persons with chronic illness are often themselves older adults. A review [21] identifies facilitators for adoption of telehealth among older adults that include low tech platforms (e.g. smart phones), devices that generate user friendly reminders.

Since 2009, according to the National Board of Health and Welfare in Sweden, the Social Act requires that the municipalities provide support to family caregivers of persons with chronic illness, older persons and persons with functional variation [3]. Persons with dementia are included as one of the targeted populations in this Social Act. Many of their family caregivers are digitally literate with greater exposure and use of smart phone and digital device, yet interactive and user-friendly mobile apps addressing their health are rare. Therefore, the purpose of this study is to develop and test an interactive mobile app targeted for family caregivers of PWD.

## Methods/design

The intervention project aims to assess the effects of providing professional support through a mobile app that is tailor-made for family caregivers of persons with dementia on improving their mental health and quality of life.

### Primary outcome

1. Reduction of caregiver stress of family caregivers of persons of dementia.

### Secondary outcome

1. Reduction of depressive symptoms amongst family caregivers of persons of dementia.
2. Reduction of loneliness amongst family caregivers of persons with dementia.
3. Improvement of quality of life of family caregivers of persons of dementia.

A pragmatic intervention design will be used with pre- and post-intervention assessments. The pragmatic elements assess the intervention in a real-time environment, ensuring best possible applicability and generalizability into the context in which the study takes place. The intervention is individualized according to the needs of family caregivers and not a fixed intervention that is the same for all. It also provides evidence for recommendations of further implementation of the study once the research has been evaluated [22–24].

### Study participants and inclusion criteria

*Family caregiver* (FC) in the study is defined as a person who provides informal care to a PWD living at home. Inclusion criteria of FCs include: adults who have provided care to a PWD living at home for at least 6 months, possess a smartphone or tablet, has access to the Internet at his/her own cost and able to read and write Swedish. FCs aged less than 18 years and/or those suffering from conditions that impede communication will not be included in the study.

#### Healthcare professional (HP)

HPs include nurse or nurse assistants specialized in dementia care and providing support to persons with dementia and their FCs in municipalities. The intervention will be implemented and facilitated by HPs based at the municipalities.

### Study settings

The intervention study will be conducted in municipalities in Region Stockholm and Region Västerbotten in Sweden. Region Stockholm includes the largest urban population in Sweden. Region Västerbotten is located in the north of the country.

### Sampling

Participants in the intervention study will include i) FCs of PWD living at home; and ii) HPs based at municipalities who will implement the intervention.

#### Quantitative

Sample size calculation of FCs is based on the primary outcome Caregiver’s Stress assessed by Zarit Burden Interview [25]. Assuming an effect size of 0.40, alpha .05, power 0.80, and 50% refusal or drop-out rate, the required sample size will be 78 FCs. In each study site, municipalities with HPs will be listed. HPs (approximately 5) at the randomly selected municipalities will be invited to participate in the study. FCs will be identified from the network of HPs that they provide support to for dementia care. The HPs will be responsible for coordinating 8–10 FCs in a group. If a HP provides support to more than 10 FCs, FCs will be randomly selected. Those not selected will continue to receive the standard support.

#### Qualitative

After completion of the intervention, 15–20 FCs and all HPs will be purposively selected from the two study sites for in-depth individual and focus group interviews.

### The intervention

The main thrust of the intervention is to provide support to the FCs by HPs through the interactive mobile app. The intervention will be implemented through the app over an 8-week period.

**Fig. 1:**
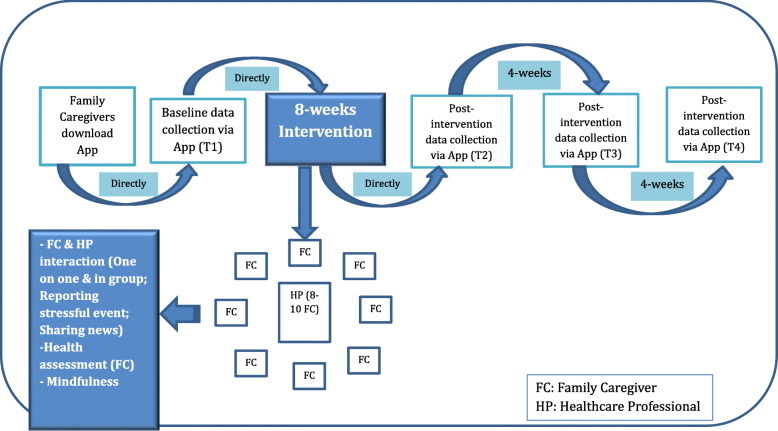
Care of Family Caregivers of Persons with Dementia (CaFCa): Research Plan

On receiving informed consent from the FCs, the selected HPs will approach them in their respective areas to download the mobile app on their smart phones or tablets (See Fig. 1). The following **functions** of the complex intervention will be conducted through the mobile app:

1. **Baseline health assessments** will be conducted via the mobile app. For baseline assessment, the FCs will be requested to go through the health assessment tools by answering questions in the app right after they download the app.
2. **Interaction with HP**: FCs will be able to communicate directly with the HP through the app on a one-on-one basis. In addition, the HP will use a predetermined protocol to moderate group discussions on a weekly basis among a group of FCs.
3. **Peer support:** During the 8-week period, the FCs will be able to communicate with their peers, i.e. other FCs of PWD, at the time of their preference.

The above functions can be conducted through the following **features** of the mobile app:

1. ***Health assessment tool*:** FCs will be able to conduct self-assessments of stress, depressive symptoms, loneliness, quality of life and self-rated health. All data from the app will be secure and accessed solely by the research team.
2. ***Chat*:** FCs will be able to communicate with other FCs for peer support and directly with HPs via the app, exchanging information and ideas on how to address the challenges in caring for a PWD, and where FCs can get support in their neighborhood.
3. ***Diary*:** FCs document any events that may be stressful or upsetting for them, such as behavioral changes in the PWD and the FCs’ responses in order to seek support from the HPs.
4. ***Mindfulness:*** Mindfulness exercises will be available on the app for the FCs.
5. ***Notifications*:** The HPs can share relevant information and events with the FCs.

The HPs will be trained by members of the research team including nurses with expertise in care of older persons and a psychologist to implement the intervention. The themes of the weekly group discussions are identified based on the findings of the qualitative interviews with FCs and HPs. These include physical and behavioral changes in dementia such as personality changes, progression of the disease, pain; importance of the FCs taking time for themselves e.g. for leisure activities, rest and sleep; complexity of feelings as a family member of a PWD, e.g. grief, shame, love; change in role from a partner or a child or a close relative of the PWD to a caregiver; financial and legal matters related to dementia care; and exploring the way forward.

Moderated by the research team, the HPs will meet twice during the intervention period to discuss and calibrate their approach to the group discussions, and to discuss challenges in leading the discussions. This will enhance fidelity of the intervention.

### Data collection

The *quantitative data* will be collected through the mobile app to capture the impact of the intervention implemented by HPs in alleviating stress, depressive symptoms and loneliness, and improving quality of life of the FCs.

The intended health related outcomes of the intervention study are presented in Table 1. Background information of the FCs such as demographic and living arrangement of the PWD cared for will be included. Outcomes will be measured via ***Health Assessment Tool*** in the app at baseline (T1) and three post-intervention time-points: right after the completion of the 8-week intervention (T2), 4 weeks (T3) and 8 weeks (T4) after the intervention (See Fig. 1).

**Table 1:** Measurement of the primary and secondary outcomes in the intervention study

Primary outcome	Measurement
Caregiver stress	For caregivers of PWD: Zarit Burden Interview (12 items) [25]
Secondary outcomes	
Depressive symptoms	Patient Health Questionnaire (PHQ-9, 9 items) [26]
Loneliness	Single item question on loneliness [27]
Quality of Life	CarerQol-7D (7 dimensions) [28]

*In-depth interviews* with FCs will be conducted to get an understanding of their experiences of using the mobile app, peer support amongst the FCs and support from the HPs. The HPs will be interviewed to evaluate the implementation of the mobile app in terms of use and practicality of the app, and particularly in combination to their regular responsibilities. The interviews will be undertaken using a semi-structured interview guide and take place shortly after the 8-week intervention (T2).

Usage of the app will be assessed by analytics information, logged on backend system, on each of the features of the app mentioned above.

The usability of the intervention will be assessed by the following parameters:

1. Compliance of the usage of the app as indicated below (Derived from App data).
  - Use of health assessment tools.
  - Reporting of stressful events.
  - Frequency of communication between FC and the HP.
  - Frequency of communication between FCs (Peer support).
  - Participation of FCs in the weekly group discussions moderated by the HP.
  - Use of mindfulness exercises.
2. User Experience in terms of satisfaction level of the app interface and the content of the intervention (Interviews with FCs and HPs).
3. User Experience in terms of ease of usage of the mobile app (Interviews with FCs and HPs).

### Data analysis

The *quantitative* data will be analyzed to describe and compare differences of means and standard deviations in stress, depressive symptoms, loneliness and quality of life amongst FCs between the baseline and post-intervention phases. ANOVA will be used to identify characteristics of FCs that are associated with the outcomes. Impact of the intervention on the probability of improving the outcomes specified in Table 1 will be assessed by regression analysis. All data will be entered and analyzed using Statistical Package for Social Scientists.

The *qualitative* data will consist of transcripts from the in-depth interviews with FCs and HPs. Using content analysis [29], the qualitative data will be analyzed to explore FCs’ experience of the use of the app. HPs’ experiences will be analyzed in terms of ease of use and practicality of the app as well as its integration in the standard care that they provide.

The *logged* data on the mobile app will be quantitatively analyzed for associations between use of the mobile app and the outcome measures.

The core research group will have access to the full data set. Other members of the research team will be provided sub-sets of data relevant for their respective studies.

### Timeline

Sample recruitment for the intervention study is expected to begin in September 2020 in Stockholm and Västerbotten Regions. The intervention will be implemented and post-intervention assessments conducted phase wise during the period September 2020 – August 2021. Data analysis will begin in Autumn, 2020 and preliminary results are expected by Autumn, 2021.

### Dissemination

The research results will be disseminated through scientific publications in peer-reviewed journals. Eligibility of authorship will require direct engagement with the project in the form of study design, data collection, analysis, interpretation and/or writing of manuscripts. Scientific outputs and learning from the research project will be used as pedagogic material in the education programmes in both undergraduate nursing programme and specialist nursing programme of Older People Nursing at the researchers’ universities. Presentations in national and international conferences, seminars/webinars and workshops, and other professional and community forum will be made to reach the general audience. Communication about the intervention project will also be done on social media and professional networks, e.g. Twitter, Facebook, ResearchGate, LinkedIn.

## Discussion

The proposed intervention study builds on a feasibility study of a mobile app to support FCs who provide care to PWDs living at own home. Using qualitative interviews, the study set out to engage FCs of PWD and HPs specialized in dementia care in exploring their needs and expectations of a mobile app as support in the caregiving role by the former. The main areas of support that were desired were tools to simplify life and create relevant contact networks [Unpublished]. The functions that were highlighted included dialogues which could be experienced as supportive with HPs and peers in their caregiving role for a PWD. It also included easily accessible information of the relevant available health care and social services for dementia care, such as financial and legal services at the municipalities. The need for brief relaxing exercises directed to the FCs themselves, such as mindfulness, was also expressed. Simplicity in terms of contents of the app and its usability was a central theme that occurred in all the interviews. Some of the areas that emerged in the qualitative interviews were similar to those identified in a systematic review which concluded the need for high-quality evaluations of effectiveness of interventions to support FCs of PWD [30].

Digital literacy amongst the population is rapidly expanding in Sweden and globally. This interactive mobile-based intervention will make it possible to extend nursing support to FCs irrespective of their geographic distance to the nearest health care or social service center. This also provides a platform for the technology enabled FCs to alleviate stress, reduce loneliness and improve quality of life through a social network system regardless of distance between FCs and prior acquaintanceship.

The SPIRIT guidelines [31] was followed to structure the study protocol (https://www.spirit-statement.org/about-spirit/). One of the strengths of this intervention study is the inclusion of qualitative interviews with HPs specialized in dementia care and FCs form the groundwork of the development of the mobile app which is the central tool of the intervention study. Both stakeholders identified common issues to be addressed through the intervention as support to FCs. A limitation of the study is that the nature of the intervention tool excludes FCs of PWD who are not comfortable in using mobile technology. Even for those who are regular users of smart phones, writing messages in a chat function to participate in discussions with HP and peers may be challenging. The proposed mobile app has a core that is the same for all but can be modified to provide similar support to other FCs providing care to persons with chronic conditions.

## Data Availability

Not applicable.

## Abbreviations

FC: Family caregiver
HP: Healthcare professional
ICT: Information and communication technology
PWD: Person with Dementia
